# Supplementary material for: Allometry reveals trade-offs between Bergmann’s and Allen’s rules, and different avian adaptive strategies for thermoregulation
Source: Nat Commun. 2023 Feb 27;14:1101. doi: 10.1038/s41467-023-36676-w (PMC9968716; doi:10.1038/s41467-023-36676-w)
Supplement: Supplementary file 1 — Supplementary Information [file 41467_2023_36676_MOESM1_ESM.pdf]

Supplementary Information

Arkadiusz Frhlich, Dorota Kotowska, Rafał Martyka &
Matthew R.E. Symonds

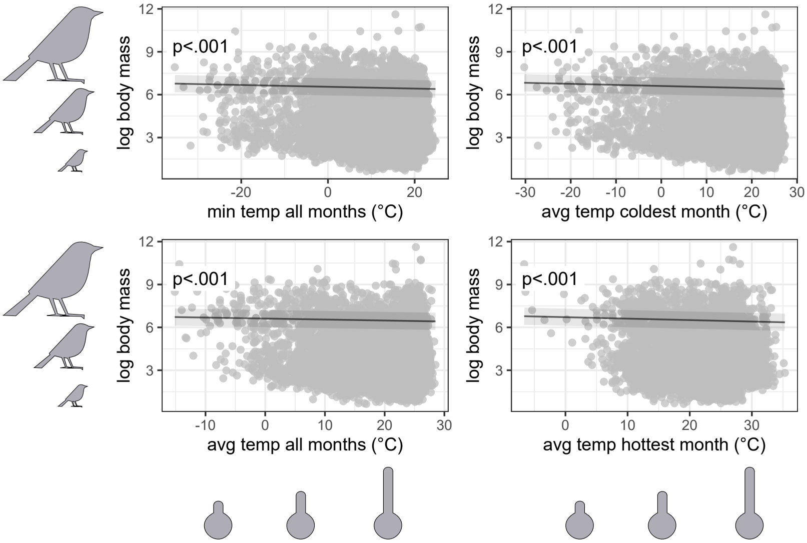

Supplementary Figure 1: Bergmann's models, showing decreasing body size with four temperature measures across 9,962 avian species (99.7% of global community). The shaded area around the trend line is simple shading to facilitate reading. The p values refer to the significance of the temperature estimates derived from two-tailed tests. See Fig. 1b in the main article for model selection procedure. The results were obtained with phylogenetic log-log linear regression by *phylolm* models on a single maximum clade credibility phylogenetic tree.

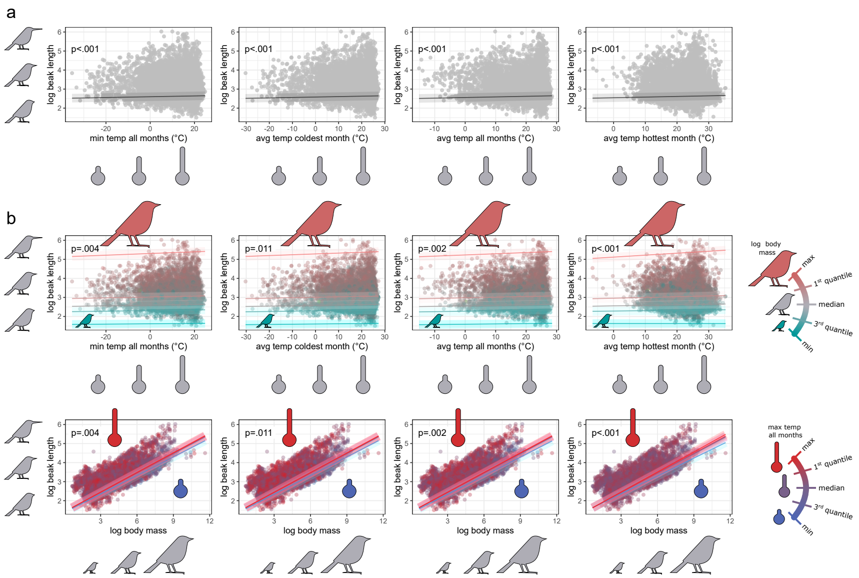

Supplementary Figure 2: The global test of Allen's rule on avian beak length across 9,962 (99.7%) species. Allen's models (a), showing the increase in avian beak length (controlled for body size, see Fig. 2a in the main article) with four temperature measures. Models with interaction of body size (allometry) and temperature (Allen's rule), illustrates how Allen's rule operates across steeping quantiles of body size (b, upper plots) and how allometry varies across steeping quantiles of temperature (b, lower plots). The p values refer to significance of temperature (a) or interaction term (b) derived from two-tailed tests. The shaded area around the trend line is simple shading to facilitate reading. See Fig. 2c in the main article for model selection procedure. The results were obtained with phylogenetic log-log linear regression by *phylolm* models on a single maximum clade credibility phylogenetic tree.

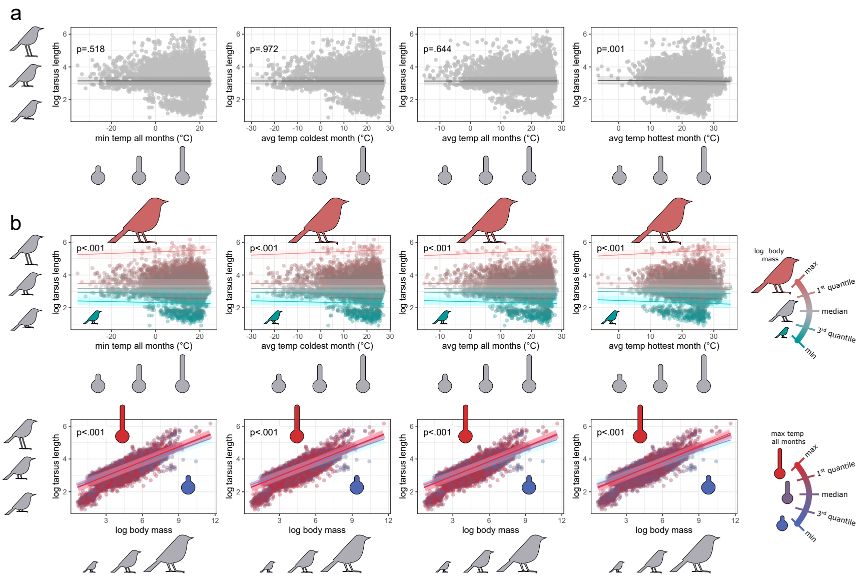

Supplementary Figure 3: The global test of Allen's rule on avian tarsus length across 9,962 (99.7%) species. Allen's models (a), showing the relationship of avian tarsus length (controlled for body size, see Fig. 3a in the main article) with four temperature measures. Models with interaction of body size (allometry) and temperatures (Allen's rule) (b), illustrates how Allen's rule operates across steeping quantiles of body size (upper plots) and how allometry varies across steeping quantiles of temperature (lower plots). The p values refer to significance of temperature (a) or interaction term (b) derived from two-tailed tests. The shaded area around the trend line is simple shading to facilitate reading. See Fig. 3c in the main article for model selection procedure. The results were obtained with phylogenetic log-log linear regression by *phylolm* models on a single maximum clade credibility phylogenetic tree.

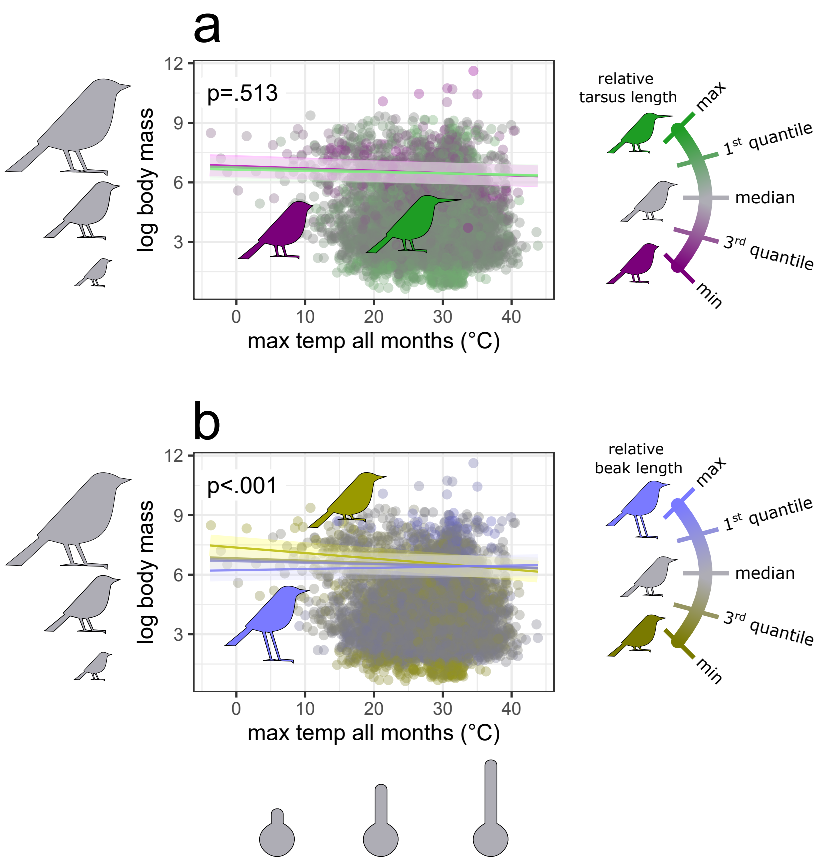

Supplementary Figure 4: Models predicting log body mass across 9,962 avian species (99.7% of global community), with interaction of relative beak (a) or tarsus (b) lengths with the max temperature of all months, illustrating how Bergmann's rule operates across steeping quantiles of relative appendage length. See Fig. 1b in the main article for Bergmann's model with no interaction with relative appendage length. The p values refer to significance of models interaction terms derived from two-tailed tests. The shaded area around the trend line is simple shading to facilitate reading. The results were obtained with phylogenetic linear regression by *phylolm* models on a single maximum clade credibility phylogenetic tree.

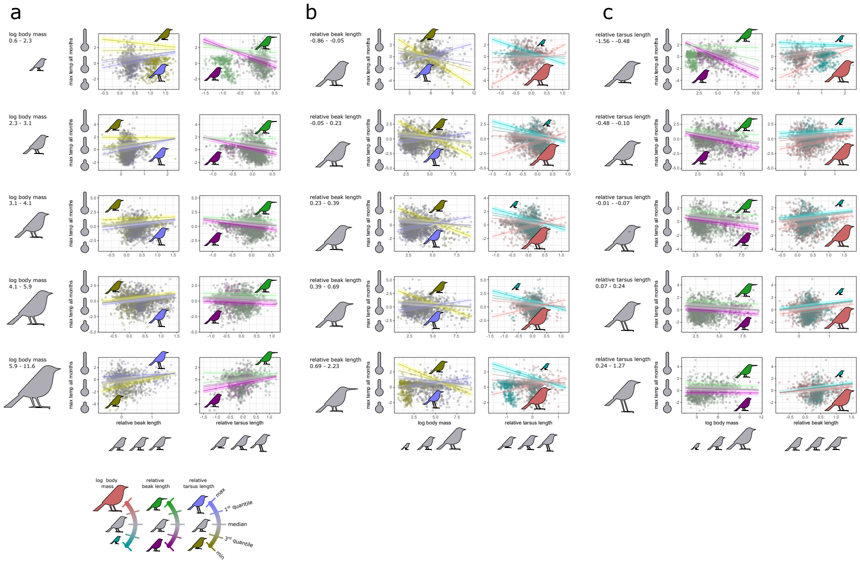

Supplementary Figure 5: Global test for avian adaptation to maximum temperature across all months by shifts in body size (Bergmann's rule) and appendage size (Allen's rule) across 9,962 (99.7%) avian species. The visualization of model with max temperature of all months as response and three-way interaction between log body mass, relative beak and tarsus lengths as predictors. Visualization (a) of the relationship between temperature and relative beak length across steeping quantiles of relative tarsus length (colors on left plots) or the relationship between temperature and relative tarsus length across steeping quantiles of relative beak length (colors on right plots), shown separately for quantiles of body size (rows). Visualization (b) of the relationship between temperature and log body size across steeping quantiles of relative tarsus length (colors on left plots) or the relationship between temperature and relative tarsus length across steeping quantiles of log body size (colors on right plots), shown separately for quantiles of relative beak length (rows). Visualization (c) of the relationship between temperature and log body size across steeping quantiles of relative beak length (colors on left plots) or the relationship between temperature and relative beak length across steeping quantiles of log body size (colors on right plots), shown separately for quantiles of relative tarsus length (rows). The model is supported by Akaike Information Criterion (ΔAIC = 0, see Fig. 4b in the main article) and the three-way interaction term is significant ($p < 0.001$, derived from two-tailed test). The shaded area around the trend line is simple shading to facilitate reading. The results were obtained with phylogenetic linear regression by *phylolm* models on a single maximum clade credibility phylogenetic tree.

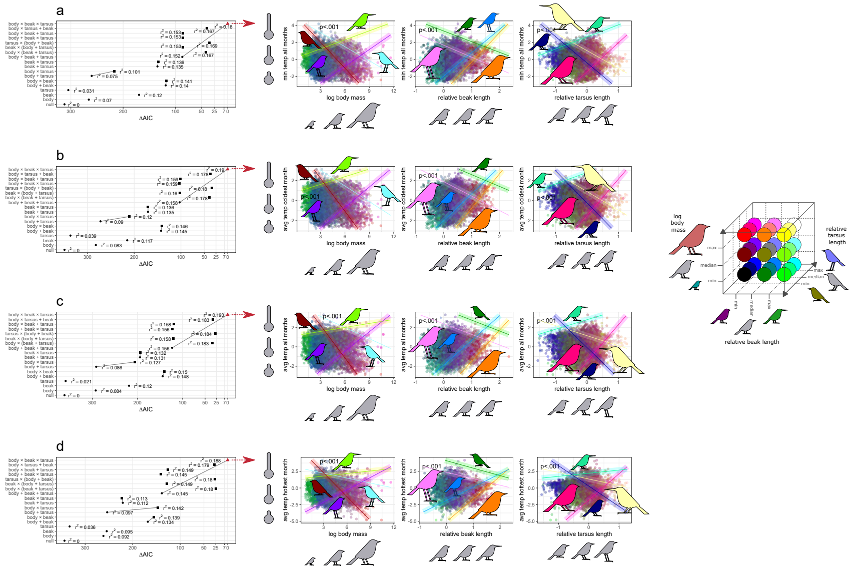

Supplementary Figure 6: Global test for avian adaptation to minimum temperature of all months (a), average temperature of the coldest month (b), average temperature of all months (c) and average temperature of the hottest month (d) by the shifts in body size (Bergmann's rule) and shape (Allen's rule) across 9,962 (99.7%) avian species. Leftmost plots show the model selection procedure including models with different combinations of log body mass, relative beak length and tarsus length as fixed and interaction terms; AIC - Akaike Information Criterion, r^2- coefficient of determination. The scatter plots on right visualize (the best) models with three-way interaction of log body mass, relative beak and tarsus length, illustrating how the temperature-based shifts in body size (Bergmann's rule) and two measures of body shape (Allen's rules) depend on each other when animals adapt to novel climates; see Fig 4f in the main article to compare these models with surrogate explaining the maximum temperature of all months. The shaded area around the trend line is simple shading to facilitate reading. The p values refer to significance of three-way interaction terms derived from two-tailed tests. The results were obtained with phylogenetic linear regression by phylolm models on a single maximum clade credibility phylogenetic tree.

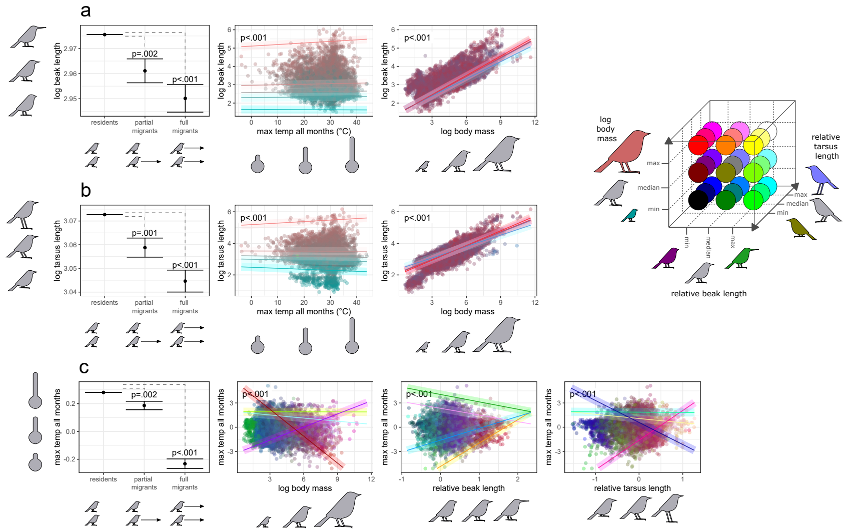

Supplementary Figure 7: The trade-offs between Bergmann's and Allen's rules across 9,962 avian species (99.7% of global community), controlled for avian migratory habits as possible confounding factor. The models predicting log beak length (a, extended from the model shown in Fig. 2e in the main article) and log tarsus length (b, extended from the model shown in Fig. 3e in the main article) with the migratory habits (categorical variable with three levels, shown on leftmost plot) and two-way interaction of log body mass and max temperature of all months (shown on two rightmost plots); trend lines illustrate how Allen's rule operates across steeping quantiles of body size (left) and how allometry of given appendage size varies across steeping quantiles of temperature (right). The model (c, extended from the model shown in Fig. 4f in the main article) predicts the max temperature of all months with the migratory habits (leftmost plot) and three-way interaction of log body mass, relative beak and tarsus length (three rightmost plots); the trend lines indicate relationships between y and x_1 (axes) across combinations of min and max values of x_2 and x_3 (colors). On leftmost plots, points indicate the model estimates, error bars indicate the confidence intervals, while p values indicate the significance of differences between resident species (n = 7,936) and partial-migrants (n = 1,109) or full-migrants (n = 917). On right plots, the p values refer to significance of two-way (a - b) and three-way (c) interaction terms. All p values are derived from two-tailed tests. The shaded area around the trend line is simple shading to facilitate reading. The results were obtained with phylogenetic linear regression by phylolm models on a single maximum clade credibility phylogenetic tree.

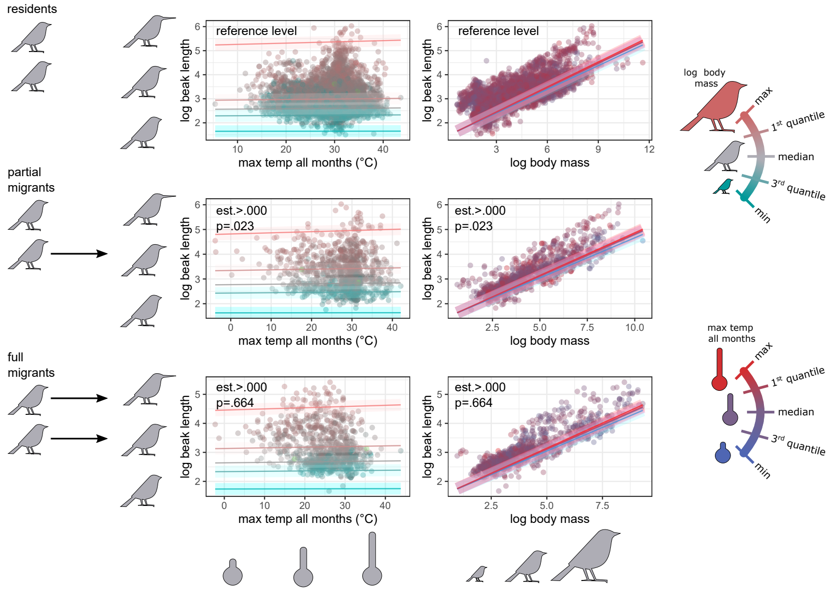

Supplementary Figure 8: The trade-offs between and Allen's rule (on avian beak length) and Bergmann's rule along the three categories of avian migratory habits across 9,962 species (99.7% of global community). The model with log beak length as response and three-way interaction of log body mass, max temperature of all months and migratory habits as predictors (extended from the model shown in Fig. 2e in the main article), visualized as the relationship between log beak length and max temperature of all months across steeping quantiles of log body mass (left plots) or the relationship between log beak length and log body mass across steeping quantiles of temperature (right plots), across categories of migratory habits (shown in rows). The pattern is slightly more prominent in partial migrants (n = 1,109) and similar in full migrants (n = 917) compared to resident species (n = 7,936) (see estimate and p values in each row; derived from two-tailed tests). The shaded area around the trend line is simple shading to facilitate reading. The results were obtained with phylogenetic linear regression by *phylolm* models on a single maximum clade credibility phylogenetic tree.

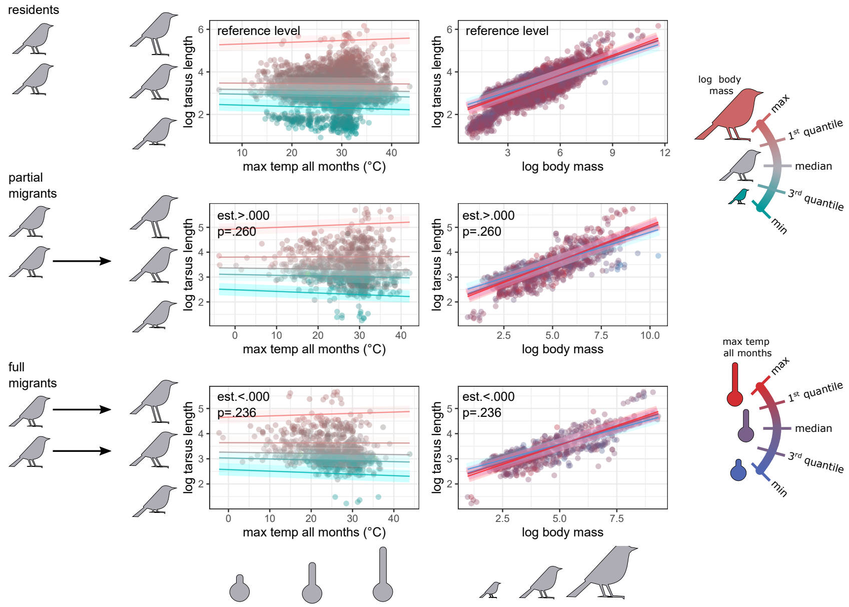

Supplementary Figure 9: The trade-offs between and Allen's rule (on avian tarsus length) and Bergmann's rule along the three categories of avian migratory habits across 9,962 species (99.7% of global community). The model with log tarsus length as response and three-way interaction of log body mass, max temperature of all months and migratory habits as predictors (extended from the model shown in Fig. 3e in the main article), visualized as the relationship between log tarsus length and max temperature of all months across steeping quantiles of log body mass (left plots) or the relationship between log tarsus length and log body mass across steeping quantiles of temperature (right plots), across categories of migratory habits (shown in rows). The pattern is similar in full migrants (n = 917) and partial migrants (n = 1,109) compared to resident species (n = 7,936) (see estimate and p values in each row; derived from two-tailed tests). The shaded area around the trend line is simple shading to facilitate reading. The results were obtained with phylogenetic linear regression by *phylolm* models on a single maximum clade credibility phylogenetic tree.

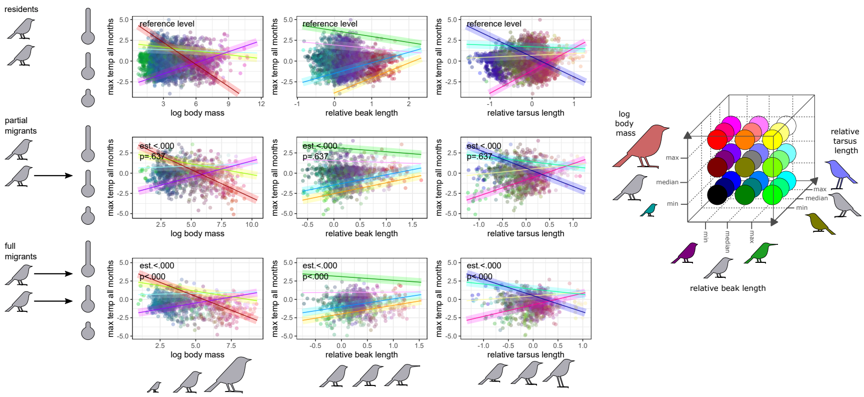

Supplementary Figure 10: Avian adaptation to maximum temperature across all months by the trade-off between shifts in body size (Bergmann's rule) and appendage size (Allen's rule) along the three categories of avian migratory habits across 9,962 avian species (99.7% of global community). The model with max temperature of all months as response and four-way interaction between log body mass, relative beak and tarsus length and migratory habits (categorical variable with three levels) as predictors (extended from the model shown in Fig. 4f in the main article). Plots show the relationship between temperature and log body mass across combinations of min and max values of relative beak and tarsus length (leftmost plots), the relationship between temperature and relative beak length across combinations of min and max values of log body mass and relative tarsus length (middle plots) and the relationship between temperature and relative tarsus length across combinations of min and max values of log body mass and relative tarsus length (rightmost plots), across migratory habits (rows). The shaded area around the trend line is simple shading to facilitate reading. The pattern is similar in partial migrants (n = 1,109) compared to resident species (n = 7,936), but is slightly less prominent in full-migrants (n = 917) compared to resident species (see estimate and p values in each row; derived from two-tailed tests). The results were obtained with phylogenetic linear regression by *phylolm* models on a single maximum clade credibility phylogenetic tree.

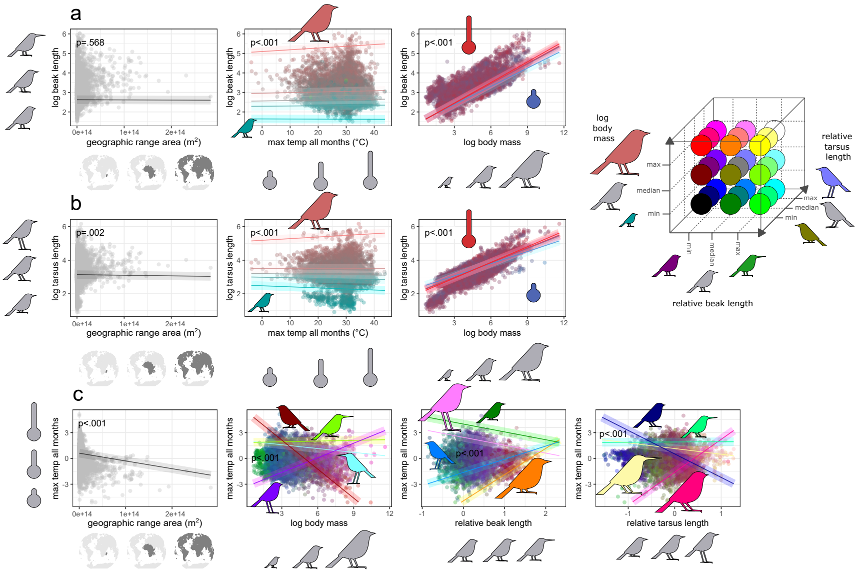

Supplementary Figure 11: The trade-offs between Bergmann's and Allen's rules across 9,962 avian species (99.7% of global community), controlled for geographic range size as possible confounding factor. The models predicting log beak length (a, extended from the model shown in Fig. 2e in the main article) and log tarsus length (b, extended from the model shown in Fig. 3e in the main article) with geographic range size (shown on leftmost plot) and two-way interaction of log body mass and max temperature of all months (shown on two rightmost plots); trend lines illustrate how Allen's rule operates across steeping quantiles of body size (middle plots) and how allometry of given appendage size varies across steeping quantiles of temperature (right plots). The model (c, extended from the model shown in Fig. 4f in the main article) predicts the max temperature of all months with the geographic range size (leftmost plot) and three-way interaction of log body mass, relative beak and tarsus length (three rightmost plots); the trend lines indicate relationships between temperature and x_1 across combinations of min and max values of x_2 and x_3 (colors). The p values refer to significance of two-way range size (leftmost plots) and three- or two-way (other plots) interaction terms derived from two-tailed tests. The shaded area around the trend line is simple shading to facilitate reading. The results were obtained with phylogenetic linear regression by phylolm models on a single maximum clade credibility phylogenetic tree.

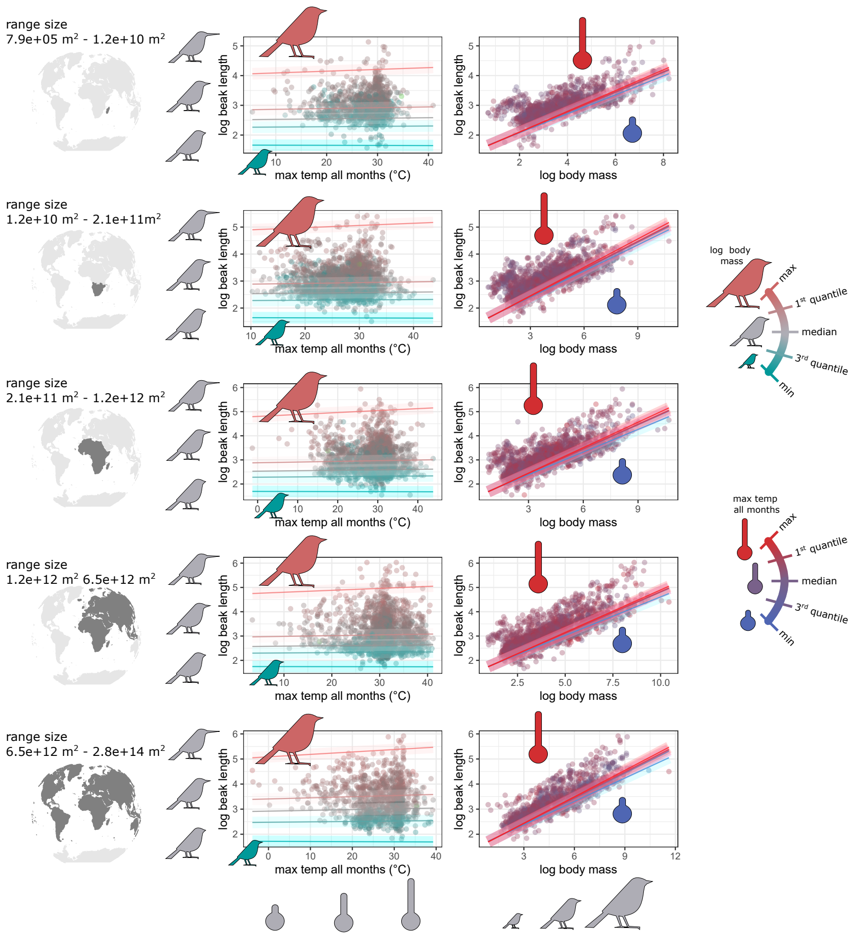

Supplementary Figure 12: The trade-offs between and Allen's rule (on avian beak length) and Bergmann's rule along the gradient of geographic range size across 9,962 species (99.7% of global community). The model with log beak length as response and three-way interaction of log body mass, max temperature of all months and geographic range size as predictors (extended from the model shown in Fig. 2e in the main article), visualized as the relationship between log beak length and max temperature of all months across steeping quantiles of log body mass (left plots) or the relationship between log beak length and log body mass across steeping quantiles of temperature (right plots), across steeping quantiles of geographic range size (shown in rows). The trade-off pattern is similar across geographic range size (three way-interaction, p = 0.415, derived from two-tailed test). The shaded area around the trend line is simple shading to facilitate reading. The results were obtained with phylogenetic linear regression by *phylolm* models on a single maximum clade credibility phylogenetic tree.

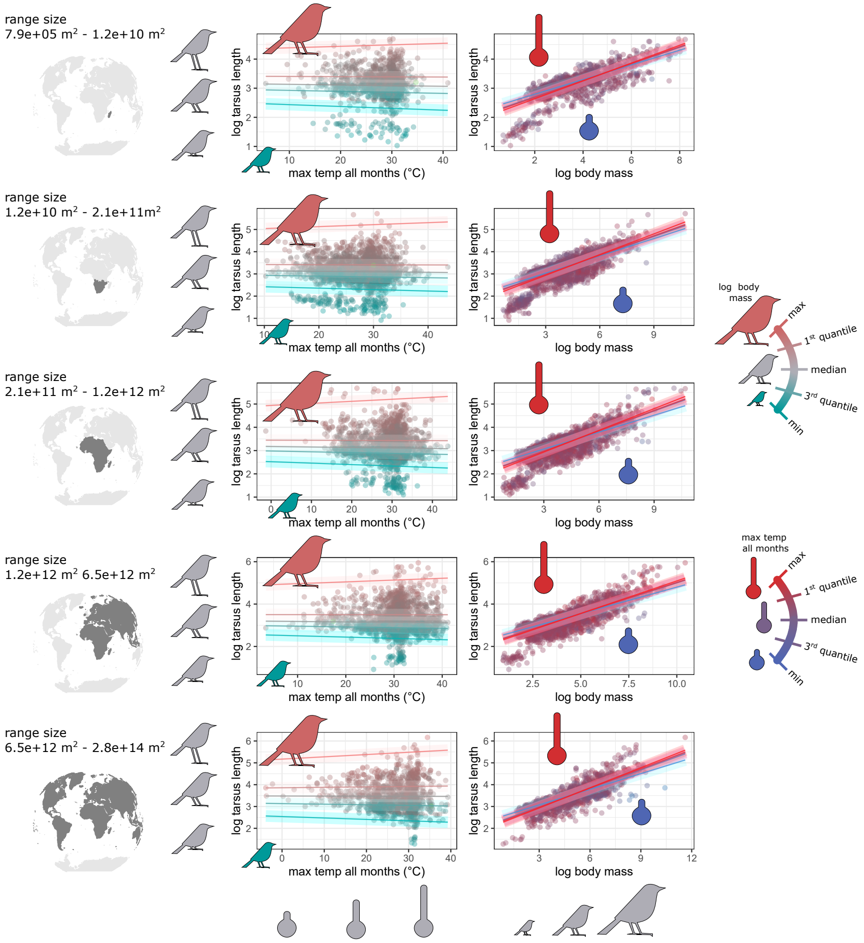

Supplementary Figure 13: The trade-offs between and Allen's rule (on avian tarsus length) and Bergmann's rule along the gradient of geographic range size across 9,962 species (99.7% of global community). The model with log tarsus length as response and three-way interaction of log body mass, max temperature of all months and geographic range size as predictors (extended from the model shown in Fig. 2e in the main article), visualized as the relationship between log tarsus length and max temperature of all months across steeping quantiles of log body mass (left plots) or the relationship between log tarsus length and log body mass across steeping quantiles of temperature (right plots), across steeping quantiles of geographic range size (shown in rows). The trade-off pattern is similar across geographic range size (three way-interaction, p = 0.583, derived from two-tailed test). The shaded area around the trend line is simple shading to facilitate reading. The results were obtained with phylogenetic linear regression by *phylolm* models on a single maximum clade credibility phylogenetic tree.

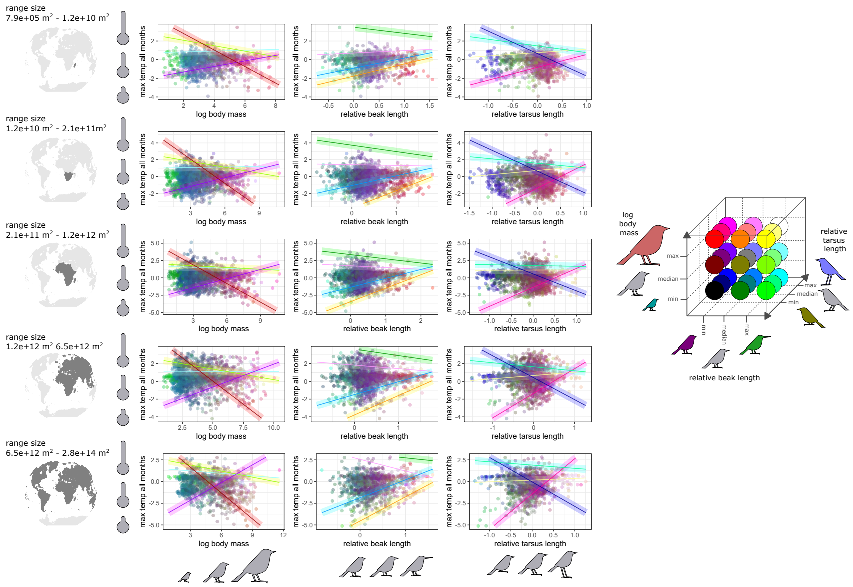

Supplementary Figure 14: Avian adaptation to maximum temperature across all months by the trade-off between shifts in body size (Bergmann's rule) and appendage size (Allen's rule) along the gradient of geographic range size across 9,962 avian species (99.7% of global community). The model with max temperature of all months as response and four-way interaction between log body mass, relative beak and tarsus length and geographic range size as predictors (extended from the model shown in Fig. 4f in the main article). Plots show the relationship between temperature and log body mass across combinations of min and max values of relative beak and tarsus length (leftmost plots), the relationship between temperature and relative beak length across combinations of min and max values of log body mass and relative tarsus length (middle plots) and the relationship between temperature and relative tarsus length across combinations of min and max values of log body mass and relative tarsus length (rightmost plots), across quantiles of geographic range size (rows). The trade-off pattern is slightly more prominent in cosmopolitan compared to endemic species (four-way interaction, p = 0.054, derived from two-tailed test). The shaded area around the trend line is simple shading to facilitate reading. The results were obtained with phylogenetic linear regression by *phylolm* models on a single maximum clade credibility phylogenetic tree.

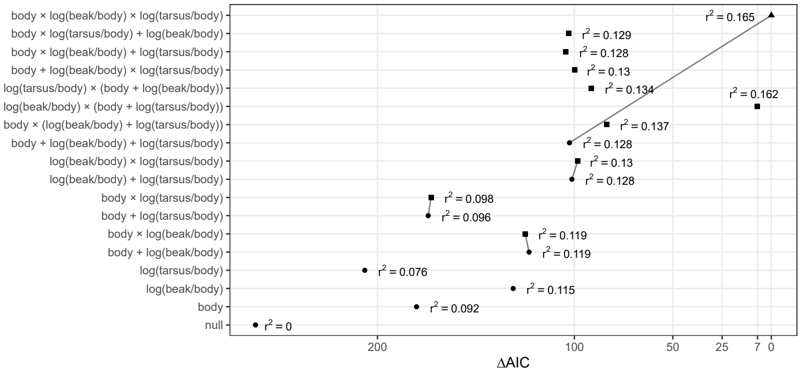

Supplementary Figure 15: Global test for avian adaptation to max temperature of all months by the shifts in body size (Bergmann's rule) and shape (Allen's rule) across 9,962 (99.7%) avian species. Model selection procedure for predicting models explaining max temperature of all months with different combinations of log body mass, log ratio of beak length to body mass and log ratio of tarsus length to body as fixed and interaction terms (surrogate of Fig 4b in the main article, with relative beak and tarsus length assessed as residuals or log-log regression); AIC - Akaike Information Criterion, r^2- coefficient of determination. The results were obtained with phylogenetic linear regression by *phylolm* models on a single maximum clade credibility phylogenetic tree.

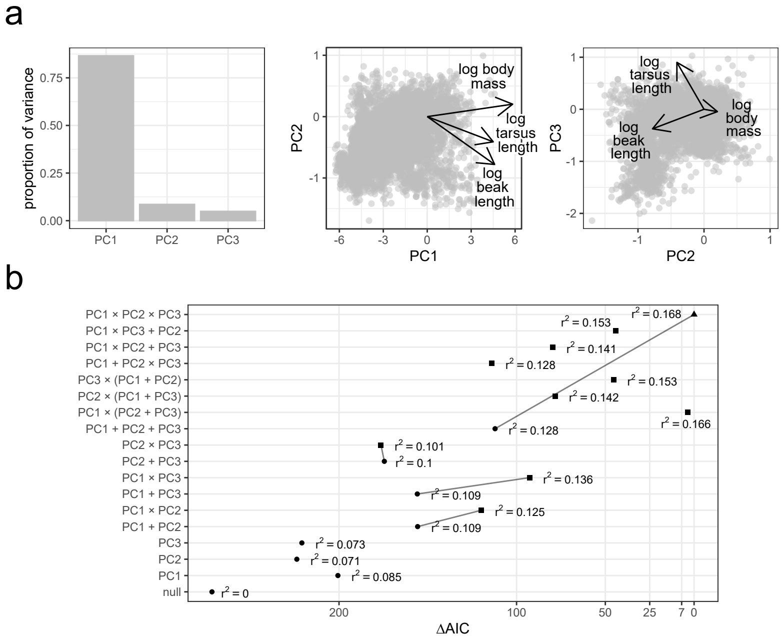

Supplementary Figure 16: Global test for avian adaptation to max temperature of all months by the shifts in body size (Bergmann's rule) and shape (Allen's rule) across 9,962 (99.7%) avian species. Phylogenetic principal component analysis of avian body size and shape (a) and the performance of different combinations of obtained components, along with their interactions (b), as predictors of max temperature of all months within avian species geographic ranges (surrogate of Fig 4b in the main article, with relative beak and tarsus length assessed as residuals or log-log regression); AIC - Akaike Information Criterion, r^2- coefficient of determination. The results were obtained with phylogenetic principal component analysis (*phytools* R package) and phylogenetic linear regression models (*phylolm* R package), using a log-transformed and scaled (mean = 0 ±1 SD) variables and a single maximum clade credibility phylogenetic tree.

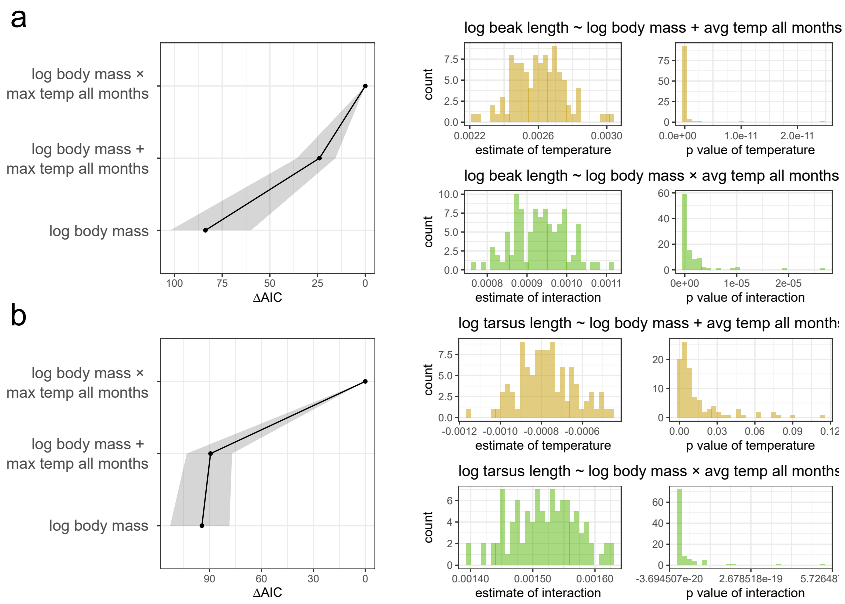

Supplementary Figure 17: The consistency of phylogenetic linear models with log-transformed beak length (a, extended from models shown in Fig. 2 in the main article) and tarsus length (b, extended from models shown in Fig. 3 in the main article) as responses, and body size and max temperature all months as either independent fixed terms or two-way interaction term, across 100 phylogenetic trees randomly downloaded from the birdTree.org website. In leftmost plots, the lines indicate the median, while ribbons indicate the range of delta Akaike Information Criterion (ΔAIC) values across set of models based on different phylogenetic trees. In rightmost plots, the histograms indicate the distribution of estimates and p values derived from two-tailed tests. Each model drawn across the same sample of 9,962 avian species (99.7% of global community).

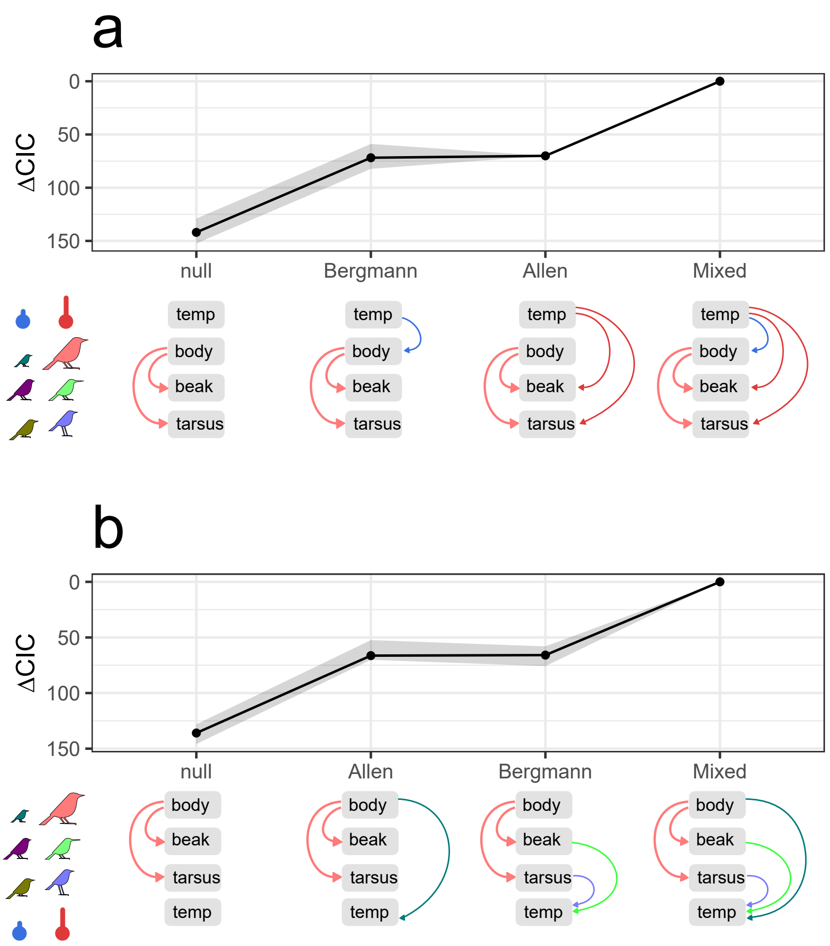

Supplementary Figure 18: The consistency of phylogenetic path analyses with log-transformed beak length and tarsus length (a, extended from models shown in Fig. 5a in the main article) and max temperature of all months (b, extended from models shown in Fig. 5b in the main article) as responses across 100 phylogenetic trees randomly chosen from the birdTree.org project. The lines indicate the median, while ribbons indicate the range of delta C statistic Information Criterion (ΔCIC) values across set of models based on different phylogenetic trees. Each model drawn across the same sample of 9,962 avian species (99.7% of global community).

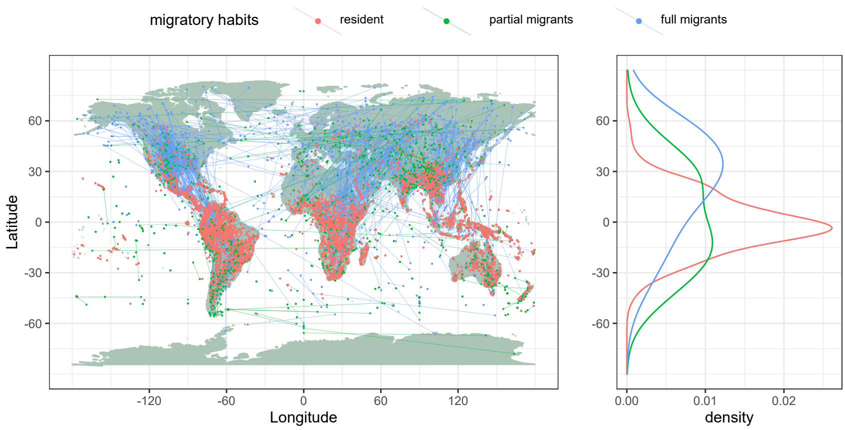

Supplementary Figure 19: Distribution of resident avian species (N = 7,936, red color), partial-migrants (N = 1,108, green color) and full-migrants (N = 917, blue color) across Earth's geographic space, shown as the centroids of geographic ranges (left plot) and the density across latitude (left plot). Note that the partial- and full- migrants include two dots (breeding and wintering ranges) connected by shadow lines, presenting their average migratory routes. Considered avian species sum up to 9,962 (99.7% of global community).

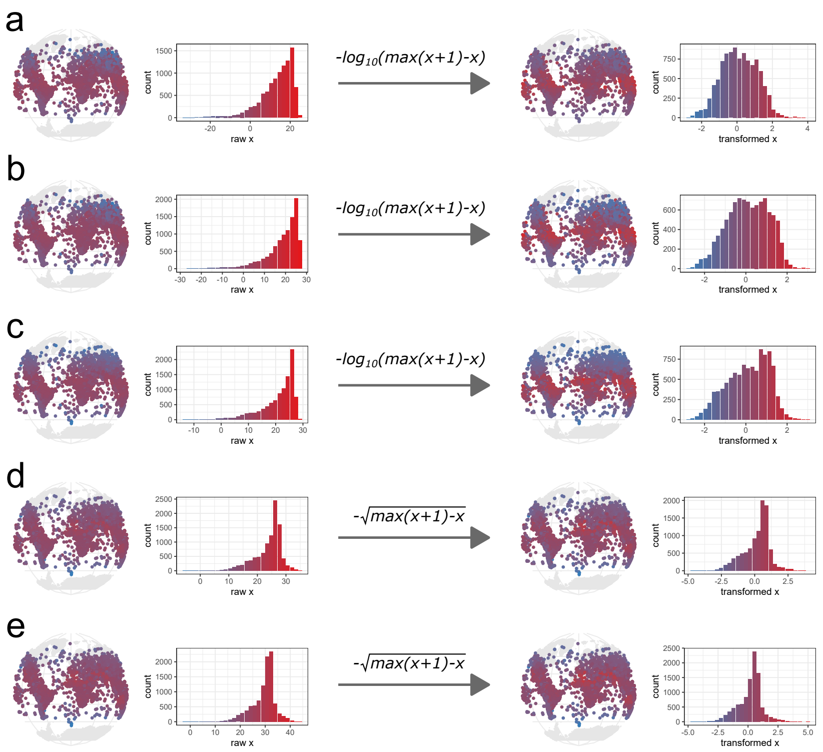

Supplementary Figure 20: The distribution of raw (left plots) and transformed (right plots) temperature variables: minimum temperature of all months (a), average temperature of coldest month (b), average temperature of all months (c), average temperature of hottest month (d) and maximum temperature of all months (e) assessed within geographic ranges of 9,962 avian species (99.7% of global community). Points on maps present geometric centroids of geographic ranges.

Supplementary Table 1: Phylogenetic signals (Pagel's λ) of phenotype and temperature variables across 9,962 avian species (99.7% of global community). Relative beak and tarsus length were obtained by retrieving residuals of log-log phylogenetic linear regression against body mass (see Fig. 2a and 3a in the main article). Temperature variables were transformed by two distinct formulas to normalize the left-skewed distributions (see Supplementary Fig. 20). The phylogenetic signals were obtained with *phylosig* function (phytools R package) on a single maximum clade credibility phylogenetic tree. The λ close to 1 means that traits are strongly predicted by phylogeny. The p values indicate the probability of rejecting the null hypothesis that $\lambda = 0$.

species' trait	Pagel's λ
log body mass	0.987 ($p<0.001$)
log (absolute) beak length	0.980 ($p<0.001$)
relative beak length	0.959 ($p<0.001$)
log (absolute) tarsus length	0.985 ($p<0.001$)
relative tarsus length	0.968 ($p<0.001$)
minimum temperature of all months	0.885 ($p<0.001$)
(transformed) minimum temperature of all months	0.852 ($p<0.001$)
average temperature of the coldest month	0.888 ($p<0.001$)
(transformed) average temperature of the coldest month	0.849 ($p<0.001$)
average temperature of all months	0.870 ($p<0.001$)
(transformed) average temperature of all months	0.854 ($p<0.001$)
average temperature of the hottest month	0.848 ($p<0.001$)
(transformed) average temperature of the hottest month	0.849 ($p<0.001$)
maximum temperature of all months	0.842 ($p<0.001$)
(transformed) maximum temperature of all months	0.843 ($p<0.001$)

Supplementary Table 2: Spearman ranks correlation matrix of temperature variables assessed within geographic ranges of 9,962 avian species (99.7% of global community). The p values refer to the significance of the correlation derived from two-tailed tests.

		(a)	(b)	(c)	(d)	(e)
minimum temperature all months	(a)		$p<0.001$	$p<0.001$	$p<0.001$	$p<0.001$
average temperature coldest month	(b)	$r_s=0.98$		$p<0.001$	$p<0.001$	$p<0.001$
average temperature all months	(c)	$r_s=0.85$	$r_s=0.89$		$p<0.001$	$p<0.001$
average temperature hottest month	(d)	$r_s=0.62$	$r_s=0.67$	$r_s=0.88$		$p<0.001$
maximum temperature all months	(e)	$r_s=0.46$	$r_s=0.55$	$r_s=0.77$	$r_s=0.92$	

Supplementary Table 3: Spearman ranks correlation matrix of temperature variables assessed within geographic ranges of 9,962 avian species (99.7% of global community). The p values refer to the significance of the correlation derived from two-tailed tests.

		(a)	(b)	(c)
log body mass	(a)		p<0.001	p<0.001
relaive beak length	(b)	r=-0.25		p<0.001
relative tarsus length	(c)	r=0.09	r=0.03	

Supplementary Note 1

```
# examplary phylogenetic linear model
library(phylolm)
linearModel1 <-
        phylolm(formula = logTarsusLength ~ logBodyMass * maxTmax,
                data = dat, phy = phylo, model = "lambda")
```

```
# examplary phylogenetic path models
library(phylopath)
modelSet <-
        define_model_set(null = c(),
                         Allen    = c(logBeakLengthCulmen ~ maxTmax,
                         logTarsusLength ~ maxTmax),
                         Bergmann  = c(logBodyMass ~ maxTmax),
                         Mixed    = c(logBodyMass ~ maxTmax,
                         logBeakLengthCulmen ~ maxTmax,
                         logTarsusLength ~ maxTmax),
                         .common = c(logBeakLengthCulmen ~ logBodyMass,
                         logTarsusLength ~ logBodyMass))
phyloPathModel <-
        phylo_path(model_set = modelSet, data = dat, tree = phylo, model = "lambda")
```
