# Supplementary material for: Allometry reveals trade-offs between Bergmann’s and Allen’s rules, and different avian adaptive strategies for thermoregulation
Source: Nat Commun. 2023 Feb 27;14:1101. doi: 10.1038/s41467-023-36676-w (PMC9968716; doi:10.1038/s41467-023-36676-w)
Supplement: Supplementary file 3 — Description of Additional Supplementary Files [file 41467_2023_36676_MOESM3_ESM.pdf]

Description of Additional Supplementary Files

File Name: Supplementary Data 1
Description: Temperature measures calculated within avian geographic ranges

File Name: Supplementary Code 1
Description: R scripts with analyses
