# Supplementary material for: Allometry reveals trade-offs between Bergmann’s and Allen’s rules, and different avian adaptive strategies for thermoregulation
Source: Nat Commun. 2023 Feb 27;14:1101. doi: 10.1038/s41467-023-36676-w (PMC9968716; doi:10.1038/s41467-023-36676-w)
Supplement: Supplementary file 6 — Reporting Summary [file 41467_2023_36676_MOESM6_ESM.pdf]

Corresponding author(s): Arkadiusz Fröhlich

Last updated by author(s): Feb 1, 2023

Reporting Summary

Nature Portfolio wishes to improve the reproducibility of the work that we publish. This form provides structure for consistency and transparency in reporting. For further information on Nature Portfolio policies, see our Editorial Policies and the Editorial Policy Checklist.

Statistics

For all statistical analyses, confirm that the following items are present in the figure legend, table legend, main text, or Methods section.

n/a	Confirmed	
☐	☒	The exact sample size (*n*) for each experimental group/condition, given as a discrete number and unit of measurement
☐	☒	A statement on whether measurements were taken from distinct samples or whether the same sample was measured repeatedly
☐	☒	The statistical test(s) used AND whether they are one- or two-sided<br>*Only common tests should be described solely by name; describe more complex techniques in the Methods section.*
☐	☒	A description of all covariates tested
☐	☒	A description of any assumptions or corrections, such as tests of normality and adjustment for multiple comparisons
☐	☒	A full description of the statistical parameters including central tendency (e.g. means) or other basic estimates (e.g. regression coefficient) AND variation (e.g. standard deviation) or associated estimates of uncertainty (e.g. confidence intervals)
☐	☒	For null hypothesis testing, the test statistic (e.g. *F*, *t*, *r*) with confidence intervals, effect sizes, degrees of freedom and *P* value noted<br>*Give P values as exact values whenever suitable.*
☒	☐	For Bayesian analysis, information on the choice of priors and Markov chain Monte Carlo settings
☐	☒	For hierarchical and complex designs, identification of the appropriate level for tests and full reporting of outcomes
☐	☒	Estimates of effect sizes (e.g. Cohen's *d*, Pearson's *r*), indicating how they were calculated

Our web collection on statistics for biologists contains articles on many of the points above.

Software and code

Policy information about availability of computer code

Data collection	Spatial analyses were carried out using 'sf' (version 1.0-8) and 'raster' (version 3.5-15) R packages and BirdLife polygon layers (version 2020.1) and WorldClim raster layers (version 2.1). Phenotypic traits came from AVONET database. Phylogenetic trees came from birdTree.org project; it was also summarized to a single consensus tree with BEAST software version 1.8.0. Data produced for the purpose of this study is made available through dryad repository (https://doi.org/10.5061/dryad.9ghx3ffn7).
Data analysis	Statistical analyses were carried out with 'phylolm' (version 2.6.2, phylogenetic linear models) and 'phylopath' (version 1.1.3, causal phylogenetic models) R packages. To plot the results we used 'ggplot2' package (version 3.3.6), implemented in custom functions that are attached as supplementary R code (https://doi.org/10.5061/dryad.9ghx3ffn7).

For manuscripts utilizing custom algorithms or software that are central to the research but not yet described in published literature, software must be made available to editors and reviewers. We strongly encourage code deposition in a community repository (e.g. GitHub). See the Nature Portfolio guidelines for submitting code & software for further information.

Data

Policy information about availability of data

All manuscripts must include a data availability statement. This statement should provide the following information, where applicable:

- Accession codes, unique identifiers, or web links for publicly available datasets
- A description of any restrictions on data availability
- For clinical datasets or third party data, please ensure that the statement adheres to our policy

Phenotype data was taken form AVONET database. Phylogenetic trees were retrieved from Bird Tree Project. Climatic data was computed using World Clim and BirdLife International datasets, and the product is now openly available in Dryad repository (https://doi.org/10.5061/dryad.9ghx3ffn7). All of this information has been incorporated into the main article.

Human research participants

Policy information about studies involving human research participants and Sex and Gender in Research.

Reporting on sex and gender	Not applicable. Human is not included in the study.
Population characteristics	Not applicable. Human is not included in the study.
Recruitment	Not applicable. Human is not included in the study.
Ethics oversight	Not applicable. Human is not included in the study.

Note that full information on the approval of the study protocol must also be provided in the manuscript.

Field-specific reporting

Please select the one below that is the best fit for your research. If you are not sure, read the appropriate sections before making your selection.

☐ Life sciences ☐ Behavioural & social sciences ☒ Ecological, evolutionary & environmental sciences

For a reference copy of the document with all sections, see nature.com/documents/nr-reporting-summary-flat.pdf

Ecological, evolutionary & environmental sciences study design

All studies must disclose on these points even when the disclosure is negative.

Study description	The study analyzes the interactions between Bergmann's rule (decrease in body size with temperature; avian body mass) and Allen's rule (increase in appendage length with temperature; avian beak and tarsus) by novel approaches to allometry and causal models
Research sample	The research sample includes 99.7% of all bird species worldwide (N = 9,962)
Sampling strategy	From the global pool of all 9,993 avian species we excluded those for which the native geographic ranges were unavailable or uncertain. This left us with 9,962 species for which we had reliable geographic data.
Data collection	Data on phenotypic traits and phylogeny were taken from open sources (see above). Temperature within species ranges has been determined by calculating the zonal statistics of the rasters of tempertaures within species ranges, using methods and sources described above.
Timing and spatial scale	Data has been collected from February to November 2022. However, variables came from open sources that include long term projects, meaning that the timing should not bias the outcomes of our analyses. Spatial bias should not imply to temperature data, as temperature was assessed within polygons covering whole species ranges. Phenotypic traits were originally collected from several specimens per species, thus should not be burdened with an error that influences the results.
Data exclusions	The research sample included 99.7% of all bird species worldwide (N = 9962) and no exclusions were done in any analyses.
Reproducibility	All analyses were repeated on different measures of temperatures, different measures of body size/shape and different phylogenetic trees. All results remain consistent regardless of applied approaches (this is explained in detail in the main text). These analyses may be reproduced with the code appended to a Dryad repository (https://doi.org/10.5061/dryad.9ghx3ffn7, but the link will become active after article is accepted).
Randomization	Not applicable. All analyses in this study are done on 99.7% of bird species.
Blinding	Not applicable. The study did not include experiments. The phenotype, phylogenetic, geographic and climatic data came from open

Blinding

sources (the authors of which were not associated with our group and were not informed on our hypotheses). The data we generated for the purpose of this study (consensus phylogenetic tree, relative appendage sizes, climatic variables for avian species) was obtained with automatic procedures, withouth any need of manual choices, thus excluded a possibility of subjective decisions to be made.

Did the study involve field work? ☐ Yes ☒ No

Reporting for specific materials, systems and methods

We require information from authors about some types of materials, experimental systems and methods used in many studies. Here, indicate whether each material, system or method listed is relevant to your study. If you are not sure if a list item applies to your research, read the appropriate section before selecting a response.

Materials & experimental systems

n/a	Involved in the study
☒	☐ Antibodies
☒	☐ Eukaryotic cell lines
☒	☐ Palaeontology and archaeology
☒	☐ Animals and other organisms
☒	☐ Clinical data
☒	☐ Dual use research of concern

Methods

n/a	Involved in the study
☒	☐ ChIP-seq
☒	☐ Flow cytometry
☒	☐ MRI-based neuroimaging
